# Sequential Transformation of Polycythemia Vera to Myelofibrosis and KMT2A-Rearranged Acute Myeloid Leukemia Treated With Revumenib: A Rare Case of Clonal Evolution

**DOI:** 10.7759/cureus.105413

**Published:** 2026-03-17

**Authors:** Christopher M Ahmad, Rae-Anne Kastle, Lara Zargarian, Yasmeen Sawalha, Samir Dalia

**Affiliations:** 1 Internal Medicine, Kansas City University, Joplin, USA; 2 Internal Medicine/Hematology, Mercy Hospital Joplin, Joplin, USA

**Keywords:** acute myeloid leukemia, jak2 mutation, kmt2a protein, leukemic transformation, menin inhibition, polycythemia vera

## Abstract

Transformation of polycythemia vera (PV) into post-polycythemic myelofibrosis (MF) and subsequently acute myeloid leukemia (AML) represents one of the most aggressive trajectories among myeloproliferative neoplasms (MPNs). Post-MPN AML carries a median survival of approximately six months, particularly among older adults with adverse cytogenetics. We report the case of a 73-year-old man with JAK2-positive PV diagnosed in 2020 who progressed to MF in 2024 and developed AML later that year. Cytogenetic analysis revealed a KMT2A (11q23) rearrangement with KMT2A::ELL fusion, a finding rarely described in secondary AML arising from MPNs. Due to advanced age, transfusion dependence, and significant cardiovascular comorbidities, the patient was not a candidate for intensive chemotherapy or hematopoietic stem cell transplantation. He was initially treated with azacitidine and venetoclax but demonstrated disease progression. In the setting of a KMT2A::ELL fusion, therapy was transitioned to the menin inhibitor revumenib, resulting in short-term clinical stability and tolerability under continued supportive care.

## Introduction

Polycythemia vera (PV) is a chronic myeloproliferative neoplasm (MPN) characterized by sustained erythrocytosis and clonal hematopoiesis, most commonly driven by JAK2 mutations [1]. While many patients experience prolonged survival, a subset develops clonal evolution leading to post-polycythemic myelofibrosis (MF) and, less frequently, acute myeloid leukemia (AML) [2]. Leukemic evolution occurs in approximately 2-5% of cases by 15 years, and the risk of transformation increases with disease duration, advancing age, and cumulative genetic mutations [3-5]. The risk of leukemic transformation increases in patients with an additional mutation or deletion in the TP53 gene [6]. While these mutations are commonly implicated, rearrangements involving KMT2A are distinctly uncommon in this setting, measuring approximately 10% of acute leukemias [7]. KMT2A rearrangements define a biologically aggressive AML subtype driven by transcriptional dysregulation, and post-MPN AML typically harbors a dismal prognosis [7]. Survival from leukemic transformation has a median of six months [4].

Menin proteins, encoded by the Men1 gene, are closely associated with MPN transformation to acute leukemia [8]. In fact, a study by Yokoyama et al. showed that the KMT2A (previously known as mixed-lineage leukemia protein) oncogenes require tight association with menin proteins for oncogenic transformation and that blasts lacking the Men1 gene were unable to undergo leukemic transformation [8]. Menin inhibitors have emerged as targeted agents for KMT2A-rearranged and NPM1-mutated AML, offering a novel therapeutic option for patients unable to tolerate intensive regimens [7]. This report describes molecular evolution from PV to MF to KMT2A::ELL-rearranged AML and illustrates the clinical application of menin inhibition in a medically complex patient.

## Case presentation

A 73-year-old man with JAK2-positive PV diagnosed in 2020 was followed longitudinally by hematology. The patient was managed at Mercy Hospital Joplin in Joplin, Missouri, USA. His medical history included coronary artery disease, paroxysmal atrial fibrillation, chronic kidney disease, obstructive sleep apnea, benign prostatic hyperplasia, and type 2 diabetes mellitus. Initial management consisted of periodic phlebotomy and cytoreductive therapy with hydroxyurea, under which his hematologic parameters remained stable for several years.

In early 2024, while receiving hydroxyurea, he developed progressive anemia, thrombocytopenia, worsening fatigue, and increasing splenomegaly. Given the new-onset cytopenias, hydroxyurea was discontinued, and a bone marrow biopsy was performed. The marrow demonstrated grade 2-3 reticulin fibrosis with megakaryocytic atypia, consistent with post-polycythemic MF. He was initiated on pacritinib, a JAK2 inhibitor commonly used in patients with MF and severe thrombocytopenia, and received treatment for approximately 2.5 months prior to leukemic transformation. Over the following months, he experienced progressive pancytopenia, escalating transfusion requirements, and continued clinical decline despite discontinuation of hydroxyurea.

**Figure 1:**
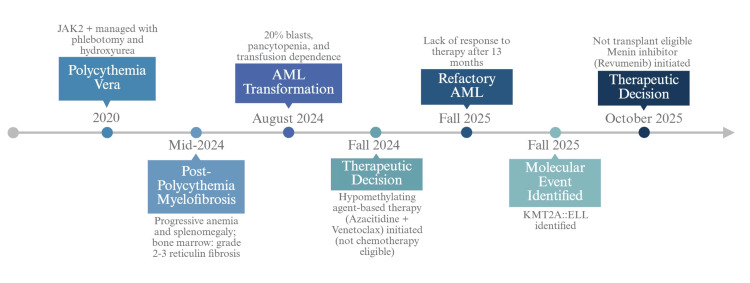
Sequential clinical and molecular evolution from PV to KMT2A::ELL-rearranged AML This original schematic illustrates the temporal progression of disease from JAK2 V617F-positive PV (2020) to post-polycythemic MF (mid-2024) and, subsequently, to AML (August 2024). The figure depicts the acquisition of >20% marrow blasts, the emergence of a KMT2A (11q23) rearrangement with a KMT2A::ELL fusion, and associated complex cytogenetic abnormalities. It further highlights therapeutic decision points, including initiation of hypomethylating agent-based therapy (azacitidine and venetoclax) and later transition to menin inhibition with revumenib. AML, acute myeloid leukemia; MF, myelofibrosis; PV, polycythemia vera This figure is an original work created by the authors using BioRender.

Due to worsening cytopenias and transfusion dependence, a repeat bone marrow evaluation was performed in August 2024 and revealed >20% myeloblasts, meeting diagnostic criteria for AML and establishing the date of leukemic transformation (Figure 1).

**Figure 2:**
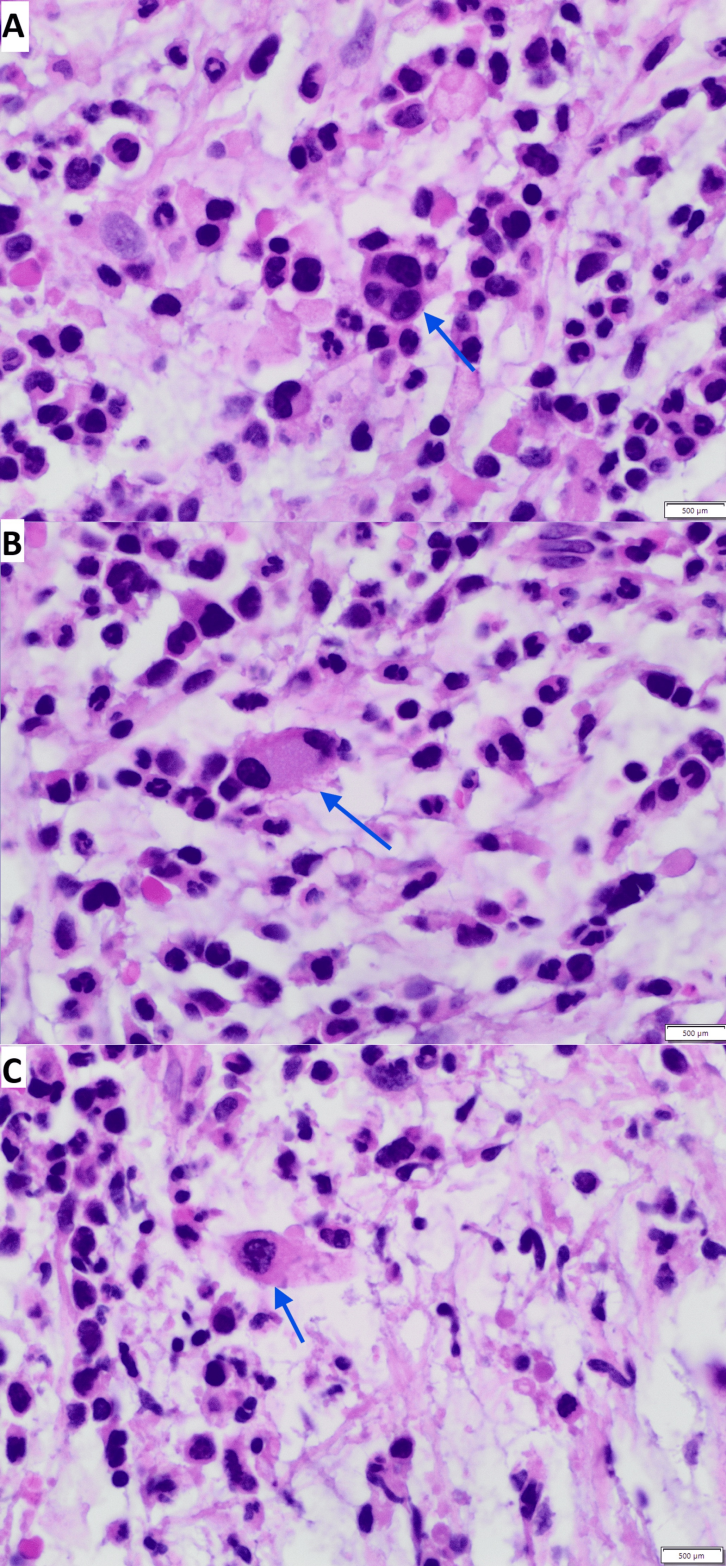
Dysplastic megakaryocytes (blue arrows) in a fibrotic bone marrow (H&E stain, 60× magnification) (A-C) High-power (60× magnification) H&E-stained bone marrow biopsy sections demonstrating atypical megakaryocytes (blue arrows). The highlighted cells exhibit hypolobated, irregularly contoured nuclei with focal hyperchromasia, consistent with megakaryocytic dysplasia. Nuclear atypia is evidenced by reduced lobulation and abnormal chromatin condensation. The surrounding marrow demonstrates increased stromal fibrosis with delicate eosinophilic fibrous strands traversing the interstitium, contributing to architectural distortion. This image represents original histopathologic material obtained from the patient described in this report. The scale bar displayed in the image corresponds to the micrometer reference embedded in the digital microscopy capture. Scale bar = 500 µm.

**Figure 3:**
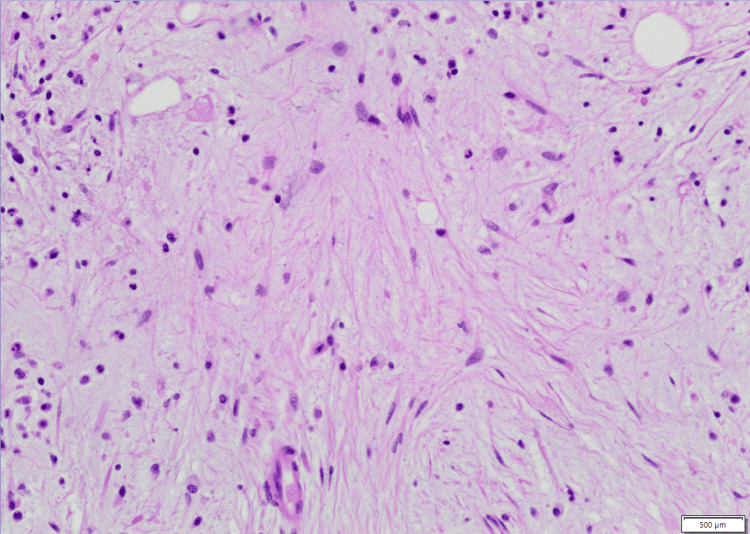
Diffuse marrow fibrosis on H&E staining (60× magnification) High-power H&E-stained bone marrow biopsy demonstrating extensive stromal fibrosis. The normal hematopoietic architecture is largely replaced by elongated, delicate eosinophilic collagen fibers traversing the marrow space. The fibrotic strands create a dense, swirling interstitial pattern with reduced cellularity and architectural distortion, consistent with advanced marrow fibrosis. This image represents original histopathologic material obtained from the patient described in this report. The scale bar visible in the image corresponds to the micrometer reference embedded in the digital microscopy capture. Scale bar = 500 µm.

**Figure 4:**
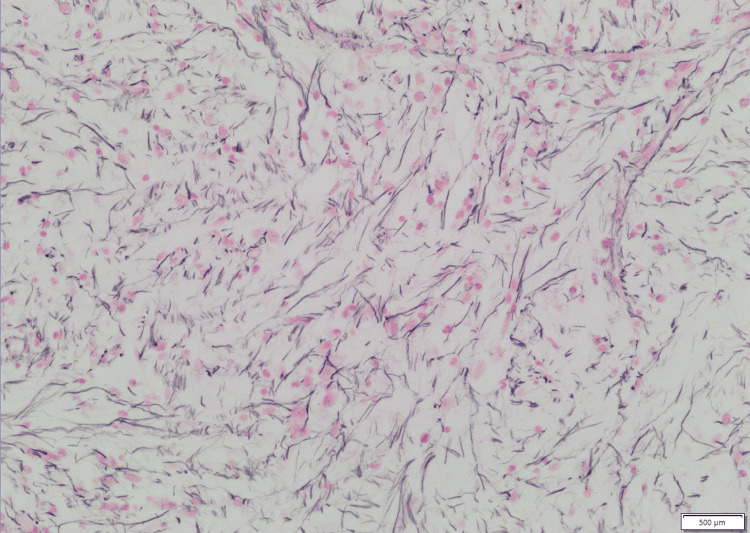
Extensive reticulin fibrosis on reticulin stain (60× magnification) Reticulin-stained bone marrow biopsy demonstrating marked reticulin fibrosis. Dense, intersecting, and branching reticulin fibers form an extensive meshwork throughout the marrow space, replacing normal hematopoietic architecture. The reticulin stain accentuates the fibrous network and is used for grading marrow fibrosis. The diffuse and confluent pattern of fiber deposition is consistent with advanced myelofibrotic involvement. This image represents original histopathologic material obtained from the patient described in this report. The scale bar visible in the image corresponds to the micrometer reference embedded in the digital microscopy capture. Scale bar = 500 µm.

Bone marrow histopathology demonstrated marked architectural distortion, extensive reticulin fibrosis, and megakaryocytic atypia. High-power examination revealed dysplastic megakaryocytes with hypolobated and hyperchromatic nuclei within a densely fibrotic stromal background (Figure 2, Figure 3). Reticulin staining confirmed advanced marrow fibrosis with intersecting fiber networks consistent with grade 2-3 involvement, as shown in Figure 4. The figures represent original diagnostic bone marrow biopsy material obtained from the patient described in this report at Mercy Hospital, Joplin.

**Figure 5:**
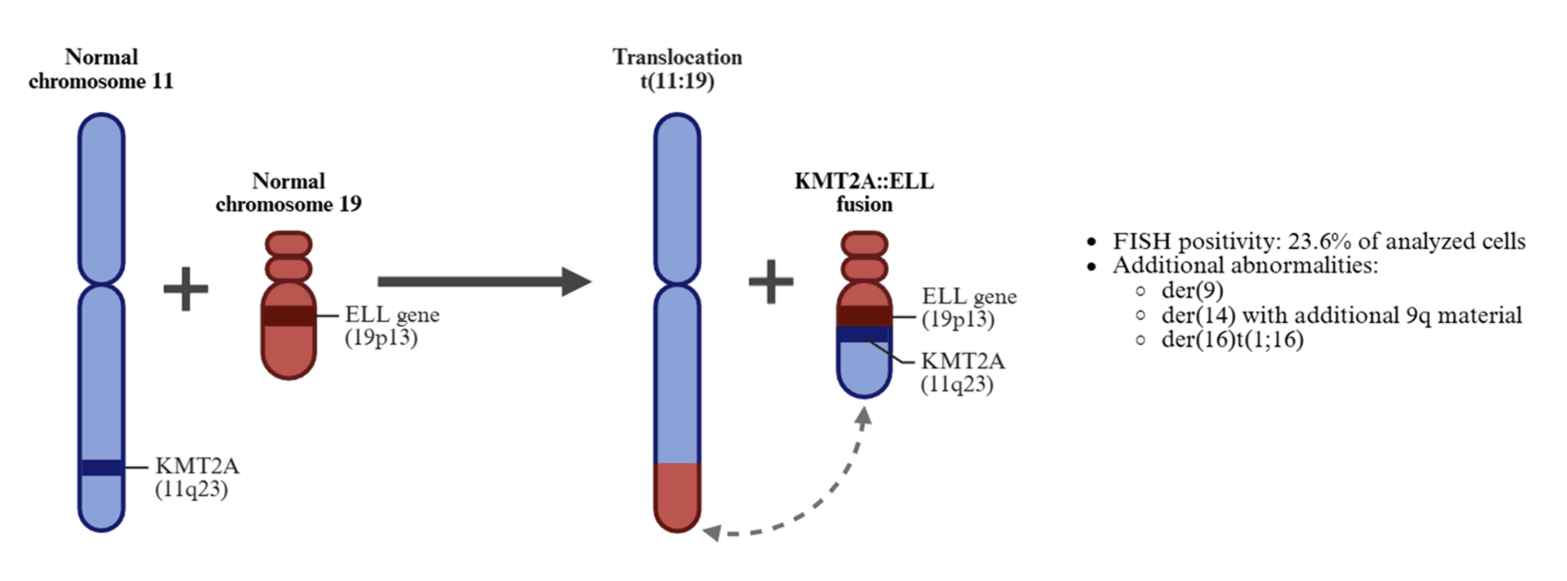
Conceptual schematic illustrating the translocation between chromosome 11q23 (KMT2A locus) and chromosome 19p13.3 (ELL locus), resulting in the formation of the KMT2A::ELL fusion gene This diagram visually represents the reported cytogenetic findings and associated structural abnormalities at AML transformation. AML, acute myeloid leukemia; FISH, fluorescence in situ hybridization This diagram is original work created by the authors using BioRender.

Cytogenetic testing performed at the University of Kansas identified a KMT2A (11q23) rearrangement. Fluorescence in situ hybridization confirmed a KMT2A::ELL fusion in 23.6% of analyzed cells (Figure 5). Additional abnormalities included a derivative chromosome 9, a derivative chromosome 14 with additional material on 9q, and a derivative chromosome 16 resulting from a 1:16 translocation. The interval from diagnosis of post-polycythemic MF (July 2024) to overt leukemic transformation (August 2024) was approximately five weeks. Laboratory evaluation demonstrated a white blood cell count of 0.9 × 10⁹/L, hemoglobin of 6.7 g/dL, platelet count of 14 × 10⁹/L, glucose of 323 mg/dL, creatinine of 0.85 mg/dL, calcium of 8.9 mg/dL, and normal liver function tests (Table 1). Electrocardiography showed sinus rhythm with occasional premature ventricular complexes and left ventricular hypertrophy criteria. The patient required weekly red blood cell transfusions.

**Table 1:** Laboratory values at AML transformation AML, acute myeloid leukemia

Parameter	Value	Reference range
White blood cells	0.9 × 10⁹/L	4.0-11.0 × 10⁹/L
Hemoglobin	6.7 g/dL	13.5-17.5 g/dL
Platelets	14 × 10⁹/L	150-400 × 10⁹/L
Glucose	323 mg/dL	70-99 mg/dL
Creatinine	0.85 mg/dL	0.6-1.3 mg/dL
Calcium	8.9 mg/dL	8.6-10.2 mg/dL

**Figure 6:**
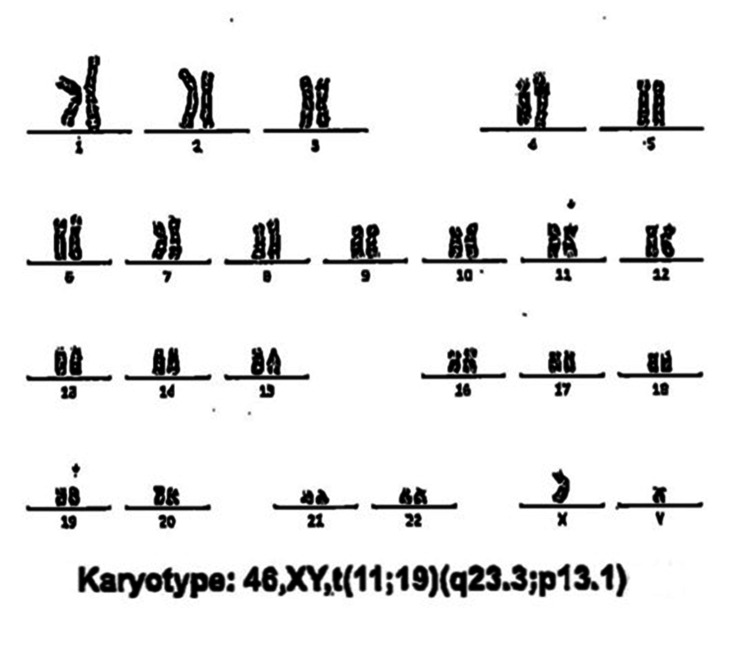
Conventional cytogenetic analysis demonstrating an abnormal male karyotype, 46,XY,t(11;19)(q23.3;p13.1), consistent with KMT2A::ELL rearrangement at AML transformation AML, acute myeloid leukemia

At the time of initial PV diagnosis in 2020, JAK2 V617F was detected; however, quantitative variant allele frequency (VAF) data from the PV and post-polycythemic MF phases were unavailable. At leukemic transformation, comprehensive myeloid next-generation sequencing confirmed the persistence of JAK2 V617F with a VAF of 42.8%, together with a GATA2 M388T variant. Because some GATA2 alterations may represent germline predisposition variants and the pathogenic significance of the M388T variant remains uncertain, this finding was interpreted as a variant of uncertain significance. RNA fusion analysis demonstrated a KMT2A::ELL rearrangement, and cytogenetic evaluation revealed an abnormal male karyotype, 46,XY,t(11;19)(q23.3;p13.1), consistent with KMT2A-rearranged AML (Figure 6). The relative stability or change in JAK2 V617F allele burden across disease phases provides insight into the clonal dynamics underlying transformation, supporting either gradual expansion of the founding clone or acquisition of a secondary leukemogenic event on a persistent MPN background.

Given advanced age, comorbid cardiovascular disease, and functional status, he was not considered a candidate for intensive induction chemotherapy or hematopoietic stem cell transplantation. Following confirmation of AML in August 2024, lower-intensity therapy was initiated in September 2024 with subcutaneous azacitidine in combination with venetoclax as first-line treatment for secondary AML. Azacitidine was administered at 75 mg/m² subcutaneously on days 1-7 of a 28-day cycle. Venetoclax was initiated with standard ramp-up dosing (100 mg on day 1, 200 mg on day 2, and 400 mg daily from day 3 onward), with administration with food. Allopurinol 300 mg daily was started three days prior to venetoclax initiation and continued throughout the tumor lysis prophylaxis ramp-up phase.

He ultimately completed approximately 13 cycles (13 months) of hypomethylating agent-based therapy prior to documented disease progression. He was initiated on pacritinib at 200 mg orally twice daily during the MF phase and received therapy for approximately 2.5 months prior to leukemic transformation.

Despite treatment, he developed relapsed disease characterized by persistent cytopenias and ongoing transfusion dependence. In the setting of a KMT2A::ELL fusion and a lack of sustained response to hypomethylating agent-based therapy, treatment was transitioned to the menin inhibitor revumenib, initiated on October 14, 2025, at a dose of 160 mg orally twice daily. At the time of follow-up in February 2026, he had remained on therapy for approximately four months.

Antimicrobial prophylaxis included fluconazole, acyclovir, and cefpodoxime. Serial electrocardiographic monitoring was performed due to the known risk of QT interval prolongation with menin inhibition, and QT intervals remained stable throughout therapy. The patient tolerated treatment without differentiation syndrome, tumor lysis syndrome, or significant non-hematologic toxicity.

At follow-up through February 2026, he remains clinically stable with persistent transfusion dependence but without progressive blast burden or new organ involvement.

## Discussion

This case illustrates a clinically instructive example of stepwise clonal evolution from PV to MF and ultimately AML. Leukemic transformation following MPNs is associated with poor outcomes, particularly in older adults with comorbid disease [9]. Molecular progression is often driven by the accumulation of additional genetic abnormalities conferring proliferative advantage and treatment resistance.

The identification of a KMT2A::ELL fusion in post-MPN AML is unusual and suggests an alternative leukemogenic pathway distinct from more commonly reported TP53- or ASXL1-driven transformations [10]. KMT2A rearrangements disrupt epigenetic regulation of HOX gene expression, promoting uncontrolled myeloid proliferation and impairing differentiation [11]. The persistence of the founding JAK2 V617F mutation alongside the KMT2A rearrangement provides further insight into whether this represents a direct linear evolution or a branching clonal event. Although definitive clonal architecture cannot be established without single-cell sequencing, the persistence of JAK2 V617F alongside acquisition of KMT2A::ELL favors a model of branching evolution with late clonal dominance.

Menin inhibitors target the critical interaction between menin and KMT2A fusion proteins, reversing oncogenic transcriptional programs. Early-phase clinical studies have demonstrated meaningful responses and acceptable tolerability, even among heavily pretreated or medically frail patients [12]. In this patient, menin inhibition allowed disease stabilization without the morbidity associated with cytotoxic therapy.

This case reinforces several practical lessons. First, molecular reassessment is essential when MPN patients develop unexplained cytopenias or transfusion dependence. Second, identification of actionable genetic alterations can meaningfully alter management even in advanced disease. Third, targeted therapy can provide durable clinical benefit and preserve quality of life in patients ineligible for curative approaches.

The rapid transformation observed in this patient is consistent with the multistep evolutionary model of MPNs, in which genomic instability and selective pressure from chronic inflammation or cytoreductive therapy drive clonal expansion [5]. Clonal hematopoiesis in PV provides a substrate for the acquisition of secondary mutations affecting epigenetic regulation, DNA repair, and transcriptional control, ultimately permitting escape from normal differentiation pathways [5,13]. Longitudinal sequencing studies have demonstrated that transformation-associated clones may be detectable years before the development of overt AML, supporting the concept of gradual molecular evolution rather than abrupt malignant conversion [13].

The molecular landscape of post-MPN AML differs substantially from that of de novo AML. Secondary AML arising from PV or MF more frequently harbors complex karyotypes and mutations in TP53, ASXL1, and RUNX1, which confer resistance to standard induction chemotherapy and are associated with inferior survival [10,13]. In contrast, rearrangements involving KMT2A are seen in ~3% of de novo AML, with a slightly higher incidence (~9%) in therapy-related AML [10,14]. The identification of a KMT2A::ELL fusion in this patient likely suggests an atypical leukemogenic trajectory, potentially reflecting a late-arising dominant clone with unique transcriptional dependencies [15].

**Figure 7:**
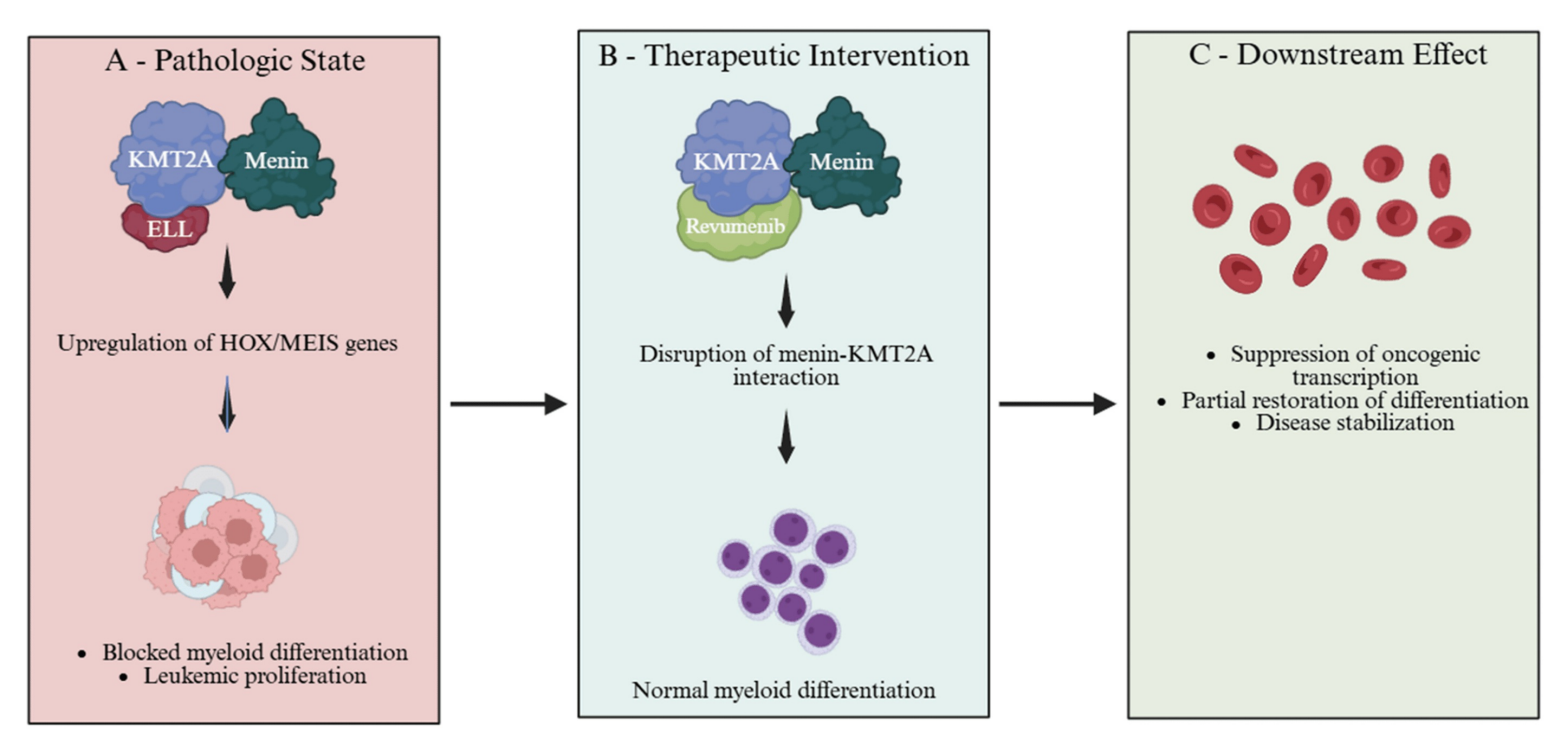
Mechanistic model of menin inhibition in KMT2A::ELL-rearranged AML This conceptual schematic illustrates the interaction between KMT2A fusion proteins and the menin cofactor at chromatin target sites, leading to transcriptional activation of HOXA9, HOXA10, and MEIS1 and impaired myeloid differentiation. The figure further depicts the mechanism of action of revumenib, which disrupts the menin-KMT2A interaction, thereby downregulating leukemogenic transcriptional programs and partially restoring myeloid differentiation. AML, acute myeloid leukemia This figure is an original work created by the authors using BioRender.

KMT2A fusion proteins exert their oncogenic effects by dysregulating HOX and MEIS gene expression, thereby impairing myeloid differentiation and sustaining proliferative signaling [11] (Figure 7). KMT2A::ELL-rearranged leukemias are characterized by aberrant transcriptional activation of HOX cluster genes, particularly HOXA9 and HOXA10, along with the transcriptional cofactor MEIS1, which together promote leukemic self-renewal and block terminal differentiation [8,11]. HOXA9 overexpression drives expansion of immature progenitors and cooperates with MEIS1 to enhance proliferative signaling, a transcriptional program strongly associated with aggressive AML phenotypes and inferior outcomes [11]. The ELL fusion partner further amplifies this oncogenic program by enhancing RNA polymerase II-mediated transcriptional elongation, stabilizing aberrant transcription complexes at HOX and MEIS loci, and amplifying downstream leukemogenic gene expression [11,16].

Unlike de novo transcriptional dysregulation observed in other AML subtypes, KMT2A fusion proteins remain critically dependent on chromatin recruitment through the menin cofactor, which anchors the fusion complex to target promoters and maintains sustained HOX/MEIS activation [8,11,13]. This transcriptional addiction to HOXA9-MEIS1 signaling creates a distinct therapeutic vulnerability, as disruption of the menin-KMT2A interaction has been shown to downregulate HOX gene expression, promote myeloid differentiation, and reduce leukemic stem cell potential in preclinical models [7,8,11]. Menin, therefore, serves as a critical adaptor protein facilitating leukemogenic transcription, and its inhibition provides a mechanistically targeted strategy to disrupt elongation-dependent transcriptional amplification without the cytotoxic effects of conventional chemotherapy [8,13].

Early-phase clinical trials of menin inhibitors in KMT2A-rearranged and NPM1-mutated AML have shown encouraging response rates, including complete remissions and meaningful reductions in blast burden among heavily pretreated patients [13,17]. The phase 2 AUGMENT-101 clinical trial of revumenib demonstrated clinically meaningful responses in patients with relapsed or refractory KMT2A-rearranged acute leukemia, further supporting the role of menin inhibition as a targeted therapeutic strategy in this molecular subtype [17]. Importantly, these agents have demonstrated a comparatively favorable toxicity profile in older adults, with manageable cytopenias and relatively low rates of non-hematologic adverse events [18]. Differentiation syndrome has been observed with menin inhibition but appears clinically manageable when recognized promptly and treated with corticosteroids [18]. In this patient, menin inhibition was introduced after progression on azacitidine and venetoclax, highlighting the importance of molecularly guided therapeutic sequencing in secondary AML.

In the present case, menin inhibition achieved disease stabilization rather than complete hematologic remission, and residual MF limited count recovery, even when the blast burden was controlled. This outcome may reflect the biological complexity of secondary AML arising from an MPN background, in which coexisting mutations and marrow fibrosis limit the full restoration of normal hematopoiesis [9]. Nevertheless, the absence of progressive leukocytosis, organ infiltration, or leukostasis over prolonged follow-up suggests effective suppression of the dominant leukemic clone [3,9]. For patients who are ineligible for intensive chemotherapy or transplantation, stabilization with preservation of quality of life represents a clinically meaningful endpoint [19].

This case further underscores the importance of repeated molecular profiling during disease progression in MPNs. Cytogenetic and next-generation sequencing reassessments can uncover therapeutically actionable alterations that would not be predicted from the original PV genotype alone [18]. Current guidelines increasingly recommend molecular testing at transformation to guide prognostication and therapeutic selection, particularly as targeted therapies become more widely available [9].

From a broader clinical perspective, this case contributes to the limited body of literature describing KMT2A-rearranged AML arising from antecedent MPNs. Most reported cases of post-MPN AML involve TP53-driven clonal evolution with extremely poor outcomes [9]. The presence of a KMT2A fusion may therefore define a biologically distinct subgroup with different therapeutic vulnerabilities, warranting further investigation [20]. Compilation of similar cases may help clarify whether menin inhibitors should be considered earlier in the treatment algorithm for selected patients with secondary AML and promote displacement of the KMT2A fusion complex from chromatin, thereby disrupting leukemogenic transcriptional programs [12].

Finally, this report underscores the growing role of precision oncology in hematologic malignancies. As molecular stratification becomes increasingly refined, treatment decisions can be based not solely on disease phase but on the dominant leukemogenic pathway driving progression [20]. In patients with limited physiologic reserve, targeted therapies may help balance disease control and tolerability, thereby extending survival while preserving functional status [19].

Limitations

This report is limited by its single-patient design, which restricts generalizability and precludes definitive conclusions regarding the efficacy of menin inhibition in post-MPN AML. The follow-up period on revumenib therapy is relatively short (four months at the time of reporting), limiting the assessment of long-term durability of response, progression-free survival, and overall survival. Additionally, serial next-generation sequencing and VAF tracking were not available to definitively characterize whether the KMT2A::ELL rearrangement arose through linear clonal evolution from the founding JAK2-mutated clone or as a branching event. Residual marrow fibrosis may have contributed to persistent cytopenias despite blast control, complicating interpretation of hematologic response. Finally, the absence of measurable residual disease monitoring further limits the precise quantification of molecular response. Larger studies with longitudinal molecular profiling and extended follow-up are needed to better define the role of menin inhibition in secondary AML arising from antecedent MPNs.

## Conclusions

Sequential progression from PV to MF and KMT2A::ELL-rearranged AML represents an uncommon and challenging clinical course. In this case, menin inhibition was associated with meaningful disease control in a medically complex, non-transplant-eligible patient. This experience highlights the importance of ongoing molecular assessment to support individualized therapeutic decision-making in patients with evolving myeloid malignancies.
